# Coinjection with TLR2 Agonist Pam3CSK4 Reduces the Pathology of Leishmanization in Mice

**DOI:** 10.1371/journal.pntd.0003546

**Published:** 2015-03-04

**Authors:** Lu Huang, Meleana Hinchman, Susana Mendez

**Affiliations:** Baker Institute for Animal Health, College of Veterinary Medicine, Cornell University, Ithaca, New York, United States of America; University of Notre Dame, UNITED STATES

## Abstract

Cutaneous leishmaniasis caused by *Leishmania major* is an emergent, uncontrolled public health problem and there is no vaccine. A promising prophylactic approach has been immunotherapy with Toll-like receptor (TLR) agonists to enhance parasite-specific immune responses. We have previously reported that vaccination of C57BL/6 mice with live *L*. *major* plus the TLR9 agonist CpG DNA prevents lesion development and confers immunity to reinfection. Our current study aims to investigate whether other TLR agonists can be used in leishmanization without induction of lesion formation. We found that live *L*. *major* plus the TLR2 agonist Pam3CSK4 reduced the pathology in both genetically resistant (C57BL/6) and susceptible (BALB/c) mouse strains. The addition of Pam3CSK4 activated dermal dendritic cells and macrophages to produce greater amounts of proinflammatory cytokines in both mouse strains. Both Th1 and Th17 responses were enhanced by leishmanization with *L*. *major* plus Pam3CSK4 in C57BL/6 mice; however, Th17 cells were unchanged in BALB/c mice. The production of IL-17 from neutrophils was enhanced in both strains infected with *L*. *major* plus Pam3CSK4. However, the sustained influx of neutrophils in sites of infection was only observed in BALB/c mice. Our data demonstrate that the mechanism behind leishmanization with TLR agonists may be very different depending upon the immunological background of the host. This needs to be taken into account for the rational development of successful vaccines against the disease.

## Author Summary

Cutaneous leishmaniasis is a skin infection caused by a protozoan parasite *Leishmania major* (*L*. *major*). The only available treatment option is chemotherapy, which is toxic and expensive. Currently, there is no vaccine. Although inoculation of virulent *L*. *major* (leishmanization) that provides effective protection in humans was widely applied, it was discontinued due to safety concerns. To improve the safety of leishmanization, we applied agonists of Toll-like receptor in the leishmanization to induce parasite-specific immune responses. In particular, we show here that inoculation with live *L*. *major* plus a TLR2 agonist Pam3CSK4 in both resistant (C57BL/6) and susceptible (BALB/c) mouse strains completely prevents the development of lesion and decreases parasite burden. The improved pathology is associated with enhanced production of IL-6 and IL-12 from dermal dendritic cells and macrophages. Both Th1 and Th17 responses are enhanced in C57BL/6 mice. Although only the Th1 response was enhanced in BALB/c mice in the presence of Pam3CSK4, there is an enhanced and sustained neutrophil influx at sites of infection. Overall, our study reveals the clinical significance of TLR2 agonist in treating cutaneous leishmaniasis. However, the protective mechanism may be quite different depending upon the genetic background of the host.

## Introduction

The prevalence of cutaneous leishmaniasis due to *Leishmania major*, a chronic disease leading to disfigurement and social stigmatization, is estimated to be at 2 million new cases each year [1]. Recent data, however, demonstrate that this number is greatly underestimated [2]. Current treatments are inadequate due to toxicity, resistance, and cost. A significant amount of work focused on prophylactic vaccine approaches have been tested in mice (*Mus musculus*), a species chosen because wild rodents are natural hosts for *L*. *major* [3]. This has included the use of attenuated parasites, parasite extracts and leishmanial antigens. Although all these vaccines have yielded promising results in rodent models [4], they have failed when tested in primates or humans [5]. Inoculation of virulent *L*. *major*, referred to as leishmanization, has been practiced in endemic areas for millennia. This practice is the only strategy that has reproducibly provided protection in humans, possibly because it mimics a natural infection, parasite persistence, and concomitant immunity. Leishmanization was widely applied, but because of exacerbated skin disease reported in rare cases [6], this strategy was discontinued. However, the traditional practice of leishmanization has made a comeback in certain endemic regions, given that it is the only vaccine with proven efficacy in humans.

Efforts to improve the safety of leishmanization have included the addition of killed parasites or immune adjuvants to reduce the size and duration of lesions [6]. Our particular approach to a safer leishmanization has been to use Toll-like receptor (TLR) agonists. TLRs are a family of 11 transmembrane proteins that specifically recognize different pathogens [7]. The therapeutic effects of TLR activation in immunotherapy are associated with the expression of high levels of IL-12 and IFN-γ In particular, the use of TLR agonists as immune adjuvants in leishmaniasis have yielded promising results. As examples, the TLR7 agonist Aldara™ showed anti-leishmanial activity in experimental models and in clinical studies of cutaneous leishmaniasis in combination with conventional therapy [8,9]. CpG DNA, a TLR9 agonist, has been extensively tested and has shown wide prophylactic and therapeutic anti-leishmanial potential [10–13]. We have previously investigated a leishmanization approach consisting of the inoculation of live parasites along with CpG DNA (Lm/CpG). We showed that Lm/CpG prevents vaccinal lesions (an undesired effect of live vaccination) in C57BL/6 mice while achieving parasite persistence and immunity [14,15]. Mechanistically, we found that Lm/CpG causes activation of dermal dendritic cells (DCs) to produce IL-6 [15] and IL-2 [16], activation of NK cells [16], and induction of Th17 response [17].

Mice have remained the major model for testing the efficacy of vaccines against cutaneous disease. Resistance or susceptibility to *L*. *major* in mice is dependent on the type of CD4^+^ helper T cell (Th) subset that is induced. Healing in resistant mice (*i*.*e*. C57BL/6) is associated with the development of IFN-γ-producing Th1 cells. In contrast, susceptibility (*e*.*g*. in BALB/c mice) is mediated by an early IL-4 production that promotes the development and expansion of Th2 cells [18]. Contrasting with these highly polarized responses in mice, human infection data show that a mixed Th1/Th2 response is more typically observed [19]. Hence, we propose that prospective prophylactic strategies must be evaluated in both Th1 and Th2 models of disease.

The aim of this study was to determine whether TLR agonists other than CpG DNA could be use in leishmanization to treat cutaneous leishmaniasis. Here, we have found that in C57BL/6 mice, *L*. *major* infection upregulates the expression of TLR2 in bone marrow-derived dendritic cells. This contrasts with our data obtained using BALB/c mice, where there is no change in the expression of TLR2 in the same cell type. Furthermore, TLR2 agonist Pam3CSK4 treatment of infected cells from both strains of mouse results in an enhanced proinflammatory response. Because TLR2 agonists have been proposed as vaccine adjuvants in other models [20–22], we investigated the use of Pam3CSK4 as an immune adjuvant in our leishmanization model. We found that leishmanization with live *L*. *major* plus Pam3CSK4 completely prevents lesion development and decreases parasite burdens in susceptible (BALB/c) and resistant (C57BL/6) mice. In both cases, dermal dendritic cells and macrophages express greater amounts of pro-inflammatory cytokines. Both Th1 and Th17 responses were enhanced in C57BL/6 mice; conversely, Th17 response was not enhanced in BALB/c mice in the presence of Pam3CSK4. However, neutrophil responses were enhanced and sustained in the susceptible mice.

## Materials and Methods

### Mice

Six-week-old C57BL/6 and BALB/c mice were purchased from Taconic and The Jackson Laboratory, respectively. All mice were maintained in the Baker Institute for Animal Health animal care facility under specific pathogen-free conditions. Animal care was in accordance with the guidelines of the Association for Assessment and Accreditation of Laboratory Animal Care, and experiments were performed with the approval of the Institutional Animal Care and Use Committee of Cornell University (Permit number: 2008–0177).

### Parasites

*L*. *major* clone V1 (MHOM/IL/80/Friedlin) promastigotes were grown at 26°C in medium 199 supplemented with 20% heat-inactivated fetal calf serum (FCS) (Gemini, Sacramento, CA), 100 U/ml penicillin, 100 μg/ml streptomycin, 2 mM L-glutamine, 40 mM HEPES, 0.1 mM adenine (in 50mM HEPES), and 5 mg/ml hemin (in 50% triethanolamine).

### Infection protocol

Infective-stage promastigotes (metacyclics) of *L*. *major* were isolated from stationary cultures (4–5 days old) by Ficoll enrichment as described before [23]. Mice were inoculated intradermally in both ears with 10^4^ *L*. *major* promastigotes alone or mixed with 50 μg (in serum free DMEM) of a single TLR2 agonist, the synthetic triacylated lipopeptide Pam3CSK4 (InvivoGen, San Diego, CA), using a 27G needle in a volume of 10 μl.

### Quantification of parasite burden

Parasite loads in the ears were determined as described previously [24]. Briefly, the ear sheets were separated and deposited in DMEM containing Liberase CI enzyme blend (0.5 mg/ml) for 60 min at 37°C. The sheets were then dissociated using a handheld tissue homogenizer. The homogenates were filtered using a 70-mm cell strainer (BD Falcon, San Jose, CA) to produce single cell suspensions and serially diluted in 96-well flat-bottom microtiter plates containing biphasic medium prepared using 50 ml Novy-MacNeal-Nicolle (NNN) medium containing 20% of defibrinated rabbit blood overlaid with 100 ml M199. The number of viable parasites in each ear was estimated by limiting dilution from the highest dilution at which promastigotes could be grown out after 7 days of incubation at 26°C. Parasite numbers were also determined in the local draining lymph node (submandibular). Lymph nodes were mechanically dissociated and parasite load was determined by limiting dilution as described above.

### Preparation of soluble *Leishmania* antigen

Thirty ml of stationary phase cultures (4–6 days old) were collected in a 50-ml tube and centrifuged at 2800 g for 15 min at 4°C. The resulting pellets were washed three times with cold 0.02 M PBS (pH 7.2) subjected to three cycles of freezing and thawing, and centrifuged at 23,000 g for 20 min. Supernatant was collected, and protein estimation was done by BCA assay following the manufacturer’s recommendations. Protein samples were stored at −80°C until use.

### Flow cytometry

Single-cell suspensions from the ear dermis were obtained as described above. For the analysis of surface markers and intracellular staining for cytokines, single cell suspensions obtained from ears and draining lymph nodes (as described above) were stimulated overnight with 25 μg/ml soluble *Leishmania* antigen, 5 ng/ml IL-2 and 10 μg/ml anti-CD28 antibody, and then cultured with brefeldin A at 10 ng/ml for 6 h and then fixed in 4% paraformaldehyde [24]. Prior to staining, cells were incubated with an anti-Fcγ III/II receptor antibody and 10% normal mouse serum in PBS containing 0.1% BSA, 0.01% NaN_3_. Cells were permeabilized and stained for the surface markers CD4 (clone RM4–5), CD11c (clone N418), Ly-6G (clone 1A8) and F4/80 (clone BM8), for the cytokines IL-6 (clone MP5–20F3), IL-12/IL-23p40 (clone C17.8), IL-4 (clone 11B11), IL-10 (clone JES5–16E3), IL-17A (clone TC11–18H10.1) and IFN-γ (clone XMG1.2). Incubations were carried out for 30 min on ice. For each sample, at least 50,000 cells were analyzed. The data were collected and analyzed using CellQuest software and a FACSCalibur flow cytometer (Becton Dickinson, San Jose, CA).

### Culture of bone marrow-derived dendritic cells and macrophages

Bone marrow-derived dendritic cells (BMDDCs) and macrophages (BMDMs) were generated as described [25]. In brief, bone marrow cells from C57BL/6 or BALB/c mice were isolated by flushing femurs and tibias with RPMI 1640. After treatment with ACK buffer to lyse red blood cells, bone marrow cells were cultured in complete RPMI 1640 supplemented with 20 ng/ml recombinant murine granulocyte-macrophage colony-stimulating factor (GM-CSF) to generate BMDDCs. Fresh cell culture medium was added on day 3 and day 6. After 9 days, floating cells were used as immature BMDDCs. For the generation of BMDMs, bone marrow cells were cultured in complete DMEM supplemented with 20% L-929-conditioned medium, which contains granulocyte colony-stimulating factor (G-CSF). Fresh cell culture medium was added on day 5. After 7 days, BMDMs were ready to use.

### *In vitro* infection of macrophages and dendritic cells and surface TLR2 expression analysis

Infective-stage promastigotes (metacyclics) of *L*. *major* from Ficoll enrichment were washed three times in PBS, resuspended at 20 × 10^6^/ml in PBS, and incubated with 5 μM 5 (6)-carboxyfluorescein diacetate succinimidyl ester (CFSE) for 15 min at 37°C. Depending on the experiment, BMDDCs or BMDMs were infected for 18 h with unlabeled or CFSE-labeled parasites at a cell/parasite ratio of 1:5. Cells were also treated with 0.5 μg/ml of the TLR2 agonist Pam3CSK4, either at the time of infection, or at 18 h post infection.

Eighteen hours post infection, supernatants were collected for cytokine analysis and cells were harvested for surface TLR2 expression analysis. To determine surface TLR2 expression, free parasites were washed away from BMDDCs culture by washing three times with cold PBS. Cells were then harvested, incubated with an anti-Fcγ III/II receptor antibody and 10% normal mouse serum in PBS, and then stained for expression of surface markers CD11c (clone N418) and TLR2 (clone 6C2).

### ELISA

Cytokine IL-12p40/p70 in the supernatants from *in vitro* stimulation was measured by sandwich ELISA as described previously [24]. All antibodies were purchased from BD Bioscience.

### RNA extraction and real-time PCR analysis

Total RNA from BMDDCs uninfected or infected with *L*. *major* was extracted using TRIzol reagent. Reverse transcription of the RNA (1 μg) was performed using SuperScript III First-Strand Synthesis System (Invitrogen, Carlsbad, CA). Real-time PCR was performed in the Applied Biosystems 7500 real-time PCR system. The reaction was performed using the FAST SYBR Green master mix (Applied Biosystems, Carlsbad, CA). Relative quantitation values were calculated using the 2^-ΔΔCt^ method. β-actin was used as the internal control for each sample. Fold changes of TLR2 mRNA were normalized to uninfected cells. The primers used were as follows: TLR2 forward, 5’-CTCTGTCATGTGATGCTTCTG-3’; TLR2 reverse, 5’-ATGTTACCCCCAGTGTCTGG-3’; β-actin forward, 5’- GCTCCGGCATGTGCAA-3’; β-actin reverse, 5’-AGGATCTTCATGAGGTAGT-3’.

### Statistical analysis

All experiments were performed two to four times with similar results. Significant differences were determined using Student *t* test or one-way ANOVA with Tukey’s post hoc test for multiple means. Statistical analysis was performed with GraphPad Prism 5 (San Diego, CA).

## Results

### *L*. *major*-infected bone marrow-derived dendritic cells upregulate TLR2 expression only in C57BL/6 mice

**Fig 1:**
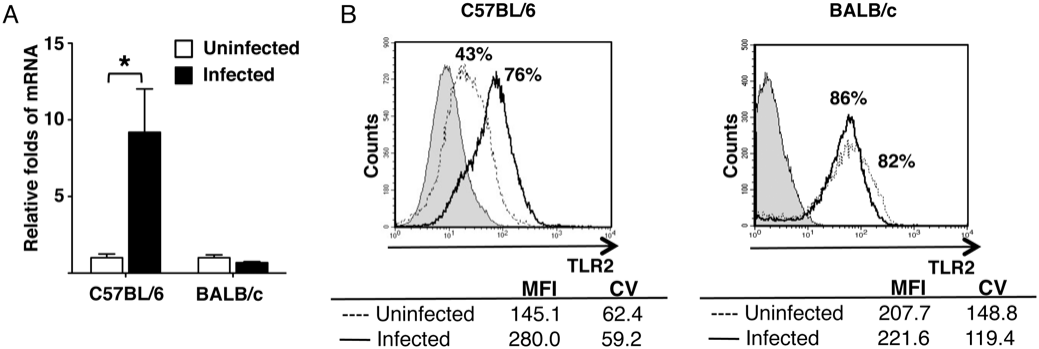
*L*. *major* infection upregulates TLR2 expression in BMDDCs from C57BL/6 mice. TLR2 mRNA transcript level measured by real-time PCR analysis, 18 h post infection (**A**). Data are normalized to β-actin, and show mean ± SD (n = 5 different experiments). Surface TLR2 expression of BMDDCs from (**B**) C57BL/6 and (**C**) BALB/c mice measured by flow cytometry. Grey histogram, unstained; dotted line, uninfected; solid line, *L*. *major*-infected cells. Mean fluorescence intensity (MFI) and Coefficient of Variation (CV) values are included in the figure. Data are representative of n = 3 experiments with similar results. Significant differences were determined by Student *t*-test. *p < 0.05.

**Fig 2:**
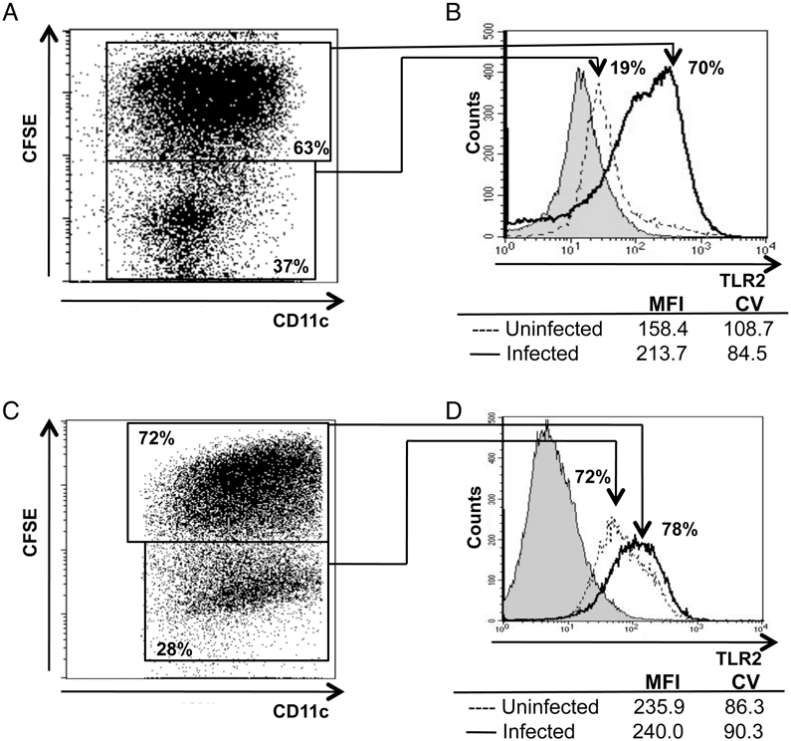
TLR2 expression is only upregulated in infected BMDDCs from C57BL/6 mice. Flow cytometry plots of bone marrow-derived CD11c^+^ DCs from (**A**) C57BL/6 and (**C**) BALB/c mice infected with CFSE-labeled *L*. *major* at a cell/parasite ratio of 1:5 for 18 h. Numbers indicate relative frequencies of infected (double positive) and uninfected (single positive) cells. Surface TLR2 expression in the infected, double positive (solid line) and in the uninfected, single positive (dotted line) populations from (**B**) C57BL/6 and (**D**) BALB/c mice. An unstained sample is included as a grey histogram. MFI and CV values are also shown. Data are representative of n = 3 experiments with similar results.

Because it has been suggested that TLR2 interacts with *L*. *major* and triggers the host immune response against the parasite [26–28], we investigated changes in TLR2 expression in *L*. *major*-infected BMDDCs. We carried out these experiments using both resistant (C57BL/6) and susceptible (BALB/c) mouse strains. TLR2 mRNA expression was upregulated 9-fold in infected DCs derived from C57BL/6 mice (Fig 1A). However, the upregulation of TLR2 was not observed in infected DCs from BALB/c mice (Fig 1A). To confirm the transcriptional results, we next determined TLR2 protein expression in both infected and uninfected BMDDCs by flow cytometry. In C57BL/6 mice, TLR2 was expressed in 43% of cells from uninfected cultures. The mean intensity of fluorescence (MFI) for the receptor was 145.1. Upon infection, the expression of TLR2 on cell surface was increased to 76%; the MFI also increased to 280 (Fig 1B). To investigate whether those changes in TLR2 expression were a direct consequence of infection, we employed CFSE-labeled parasites to directly track the infected cells. A cell/parasite ratio of 1:5 resulted in the infection of more than 60% of the cells in the culture (Fig 2A). In the uninfected cells, 19% of them expressed TLR2. However, 70% of cells containing fluorescent parasites expressed TLR2 (Fig 2B). As before, the MFI for TLR2 expression also increased in the infected cells (from 158.4 to 213.7) (Fig 2B).

Interestingly, TLR2 expression in uninfected cells from BALB/c mice was significantly higher (>80%), and infection with *L*. *major* did not significantly increase the receptor expression (Fig 1A, B). MFI values for TLR2 did not significantly change either. As expected, infection did not significantly change the already elevated expression of TLR2 (Fig 2C, D). The results suggest that infection of *L*. *major* directly induces upregulation of TLR2 only in BMDDCs from resistant (C57BL/6) mouse stain.

### Pam3CSK4 increases the ability of *L*. *major*-infected BMDDCs and BMDMs to secrete IL-12

**Fig 3:**
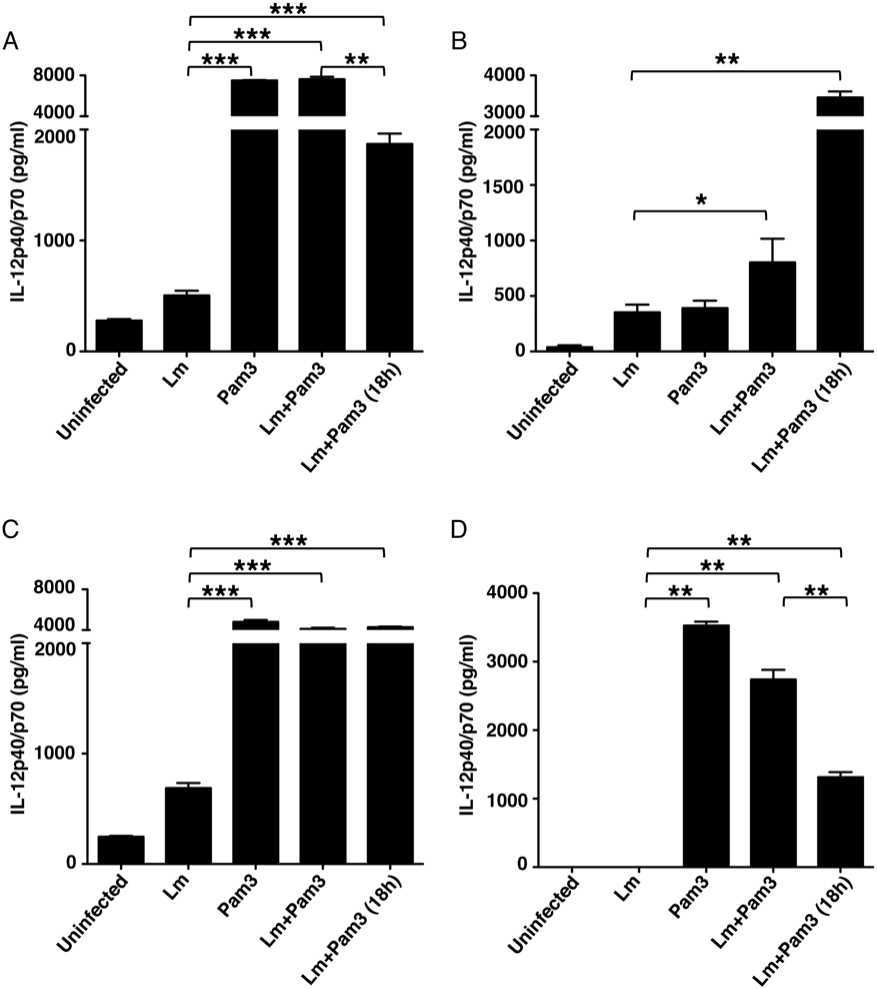
Pam3CSK4 increases secretion of IL-12 in *L*. *major*-infected BMDDCs and BMDMs. IL-12 production measured by ELISA 24 h post stimulation in culture supernatants from BMDDCs and BMDMs infected or not with *L*. *major* (cell/parasite ratio, 1:5). Infected cultures were treated with 0.5 μg/ml of Pam3CSK4 at the time of the infection (Lm + Pam3) or 18 h after the infection (Lm + Pam3 (18h)). BMDDCs data are shown in (**A**) C57BL/6 and (**C**) BALB/c mice. BMDMs data are shown in (**B**) C57BL/6 and (**D**) BALB/c mice. Data show mean ± SD (n = 5 independent experiments). Significant differences were determined by ANOVA and Tukey’s test. *p < 0.05, **p < 0.001, ***p <0.0001.

Next we determined whether the upregulation of TLR2 expression would result in an enhanced response to TLR2 stimulation. We first infected BMDDCs from both mouse strains with *L*. *major* and treated them with the TLR2 agonist Pam3CSK4, either at the time of infection, or 18 h post infection. The production of IL-12 was measured in culture supernatants 24 h post stimulation. As expected, BMDDCs from both mouse stains produce IL-12 in response to Pam3CSK4. This cytokine response was significantly greater than that secreted following infection. Interestingly, IL-12 production was enhanced in *L*. *major*-infected cells treated with the TLR2 agonist, irrespective of when it was added to the cultures in both strains (at the time or after infection) (Fig 3 A, C). We also determined the effect of agonist treatment in infected BMDMs. It is clear that *L*. *major* infection in BMDMs from BALB/c mice inhibits production of IL-12 induced by Pam3CSK4 (Fig 3D) [Pam3 vs Lm+Pam3 (18h)]. However, compared to Lm infected BMDMs, Pam3CSK4 treatment dramatically enhanced IL-12 production in BMDMs from both strains of mouse when added at the time of infection or 18 h post infection (Fig 3B, D). These results indicate that infected cells from both mouse strains are capable of responding to the TLR2 agonist stimulation.

### Leishmanization with *L*. *major* plus Pam3CSK4 prevents development of lesions and decreases parasite burdens in mice

**Fig 4:**
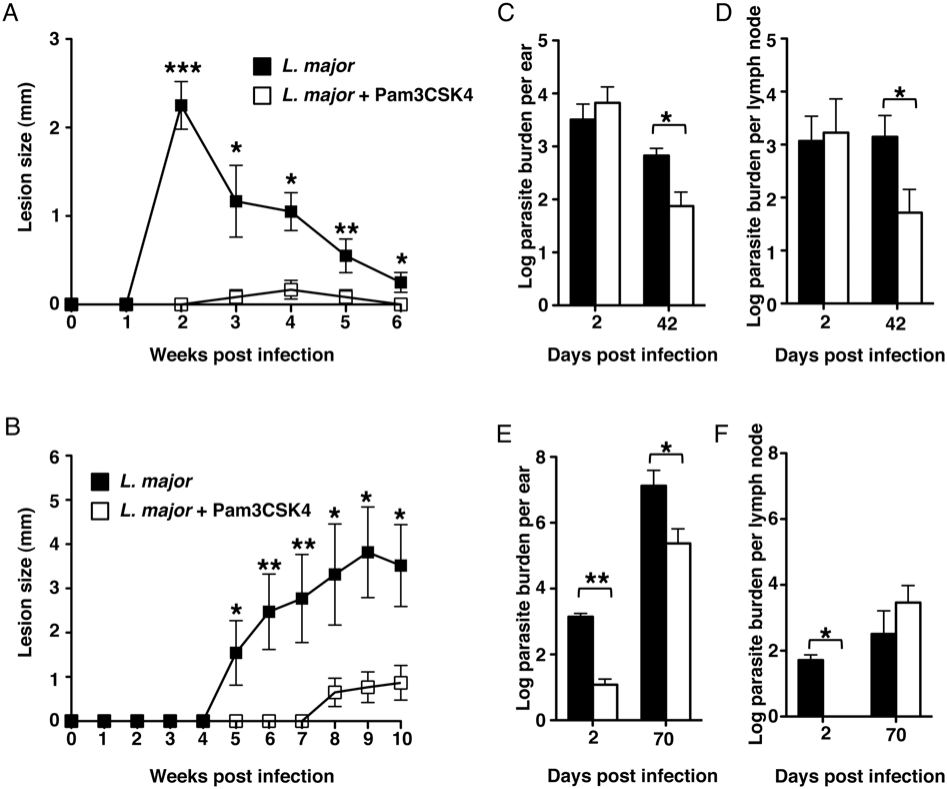
Leishmanization with *L*. *major* + Pam3CSK4 decreases lesion size and parasite burden in mice. Mice were injected in the ear dermis with 10^4^ *L*. *major* alone or in combination with 50 μg Pam3CSK4. Figure shows lesion size (mm) in (**A**) C57BL/6 and (**B**) BALB/c mice. Parasite burdens shown in (**C**) and (**E**) represent data from ears of C57BL/6 and BALB/c mice respectively. Parasite burdens shown in (**D**) and (**F**) represent data from submandibular lymph nodes of C57BL/6 and BALB/c mice respectively. Data were collected at day 2 (both strains), 42 (C57BL/6) and 70 (BALB/c) post injection. Each data set was collected from two experiments with similar results. Values represent mean ± SD. n = 3 mice. Significant differences were determined by Student *t*-test. *p < 0.05, **p < 0.001.

Our *in vitro* data strongly suggested that the proinflammatory properties of Pam3CSK4 could enhance anti-leishmanial immunity *in vivo*. In order to determine the outcomes of leishmanization with Pam3CSK4, we tested our hypothesis by using two different strains of mouse. We infected C57BL/6 mice (Th1-biased, self-healing disease) and BALB/c mice (Th2 biased, progressive disease) in the ears with a suspension of 10^4^ *L*. *major* parasites with or without 50 μg Pam3CSK4. We monitored the development of lesions and determined parasite burdens in ears at early (day 2) and late (days 42 for C57BL/6 mice and 70 for BALB/c mice) time points. Surprisingly, both mouse strains inoculated with *L*. *major* and Pam3CSK4 developed either small or no lesions compared to mice infected with parasites alone (Fig 4A, B). Parasite burden data from ears and lymph nodes of C57BL/6 mice revealed no differences between the two experimental groups at the early time point (day 2) (Fig 4C, D), suggesting that treatment with the TLR2 agonist did not interfere with parasite establishment in these mice. In contrast, parasite burden was significantly decreased in both ears and lymph nodes of C57BL/6 at day 42 (Fig 4C, D).

On the other hand, establishment of *L*. *major* infection in ears and draining lymph nodes of BALB/c mice was dramatically compromised at the early time point, as parasite burden was significantly decreased in both sites at day 2 (Fig 4E, F). At day 70, parasite burden was still significantly lower in the ears of mice treated with Pam3CSK4, but no differences were detected in their lymph nodes (Fig 4E, F). These results suggest that, while Pam3CSK4 prevents the development of pathology in both mouse strains, the kinetics and the mechanism whereby pathology is prevented may be quite different.

### Pam3CSK4 increases the production of dermal proinflammatory cytokines

We have previously shown that vaccination with *L*. *major* and CpG DNA increased the early proinflammatory cytokine production in the dermis of C57BL/6 mice [15]. To determine if this activation mechanism caused by the TLR9 agonist is shared with other TLR ligands, we investigated the expression of the proinflammatory cytokines IL-12 and IL-6 at 48 h post leishmanization, in both dermal DCs (express CD11c) and macrophages (express F4/80). The total number of cells positive for IL-12 and IL-6 staining were significantly increased at 48 h in all mice inoculated with parasites plus Pam3CSK4, irrespective of the mouse strain (Table 1). This demonstrates that leishmanization with live parasites and Pam3CSK4 also induces the early initiation of a strong proinflammatory response at the sites of infection.

**Table 1:** **Absolute number (×10** ^**4**^) of IL-12 and IL-6 producing dermal CD11c^**+**^ DCs and F4/80^**+**^ macrophages in C57BL/6 and BALB/c mice at 48 h post infection with ***L*. *major*** alone or in combination with 50 μg Pam3CSK4.

		*L*. *major*	*L*. *major*+Pam3CSK4	P values
**C57BL/6**				
**CD11c** ^**+**^ **DCs**	IL-12	7.3 ± 4.0	21.4 ± 3.9	P = 0.005
	IL-6	5.8 ± 2.6	15.7 ± 6.2	P = 0.06
**F4/80** ^**+**^ **Macrophages**	IL-12	11.4 ± 1.2	32.3 ± 4.8	P = 0.001
	IL-6	5.7 ± 2.5	11.0 ± 1.2	P = 0.03
**BALB/c**				
**CD11c** ^**+**^ **DCs**	IL-12	5.1 ± 1.3	11.3 ± 2.9	P = 0.006
	IL-6	4.5 ± 2.3	11.3 ± 2.7	P = 0.02
**F4/80** ^**+**^ **Macrophages**	IL-12	4.4 ± 1.2	13.1 ± 3.3	P = 0.01
	IL-6	6.1 ± 3.0	18.8 ± 2.4	P = 0.004

Data represent mean ± SD. n = 3 mice. Significant differences were determined by Student *t* test. P values obtained from comparing both groups are included in the table.

### Leishmanization with *L*. *major* plus Pam3CSK4 induces the expansion of Th1 and Th17 cells in C57BL/6 mice

**Fig 5:**
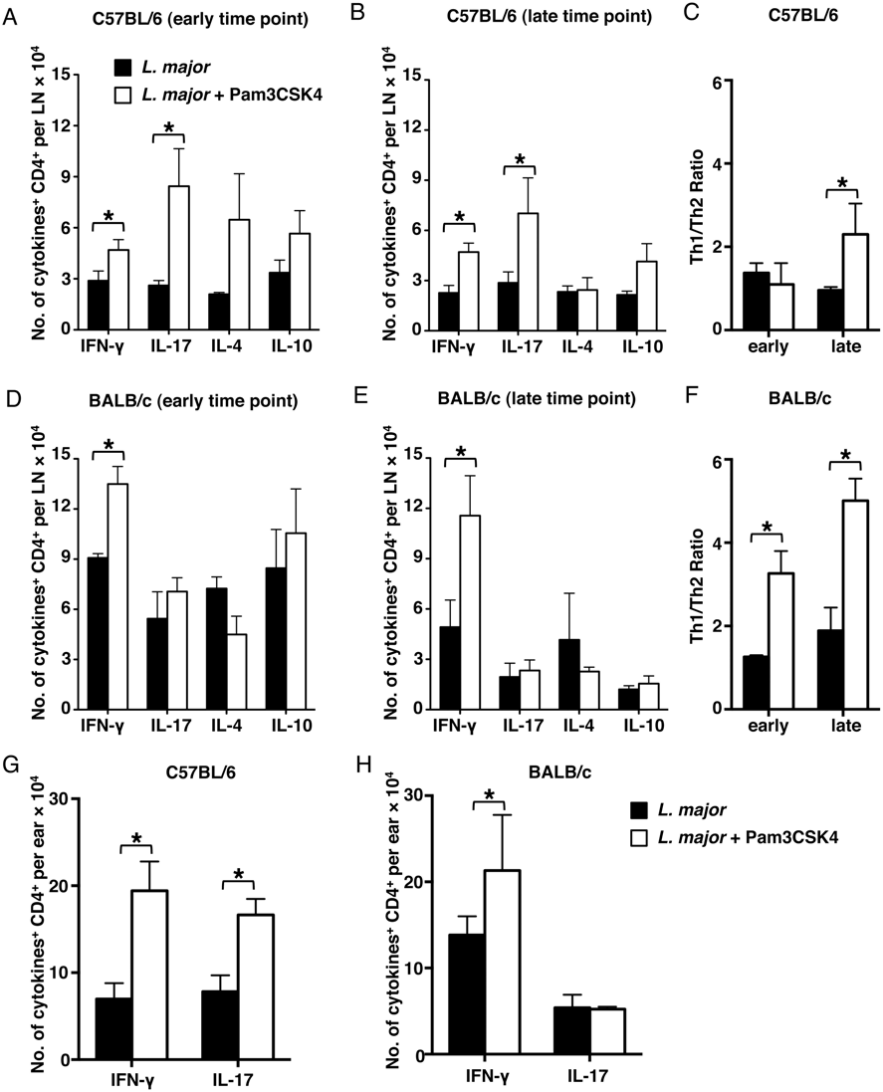
Leishmanization with *L*. *major* + Pam3CSK4 enhances Th1 and Th17 responses in C57BL/6 mice but only the Th1 response in BALB/c mice. Absolute number of IFN-γ (Th1 cells), IL-17 (Th17 cells), IL-4 (Th2 cells) and IL-10 producing CD4^+^ T cells shown in (**A**) and (**B**), (**D**) and (**E**) represents data from submandibular lymph nodes of C57BL/6 and BALB/c mice respectively. Th1/Th2 ratio is calculated as the ratio of IFN-y/IL-4 in C57BL/6 **(C)** and BALB/c **(F)** mice. Absolute number of IFN-γ and IL-17 producing CD4^+^ T cells shown in (**G**) and (**H**) represents data from ears of C57BL/6 and BALB/c mice respectively at late time point. Recovered lymph node and ear cells were restimulated with 25 μg/mL soluble *Leishmania* antigen, 5 ng/mL IL-2 and 10 μg/mL anti-CD28 overnight before performing cytokine staining. Data were collected at day 2 (both strains), 42 (C57BL/6) and 70 (BALB/c) post injection. Each data set was collected from two experiments with similar results. Values represent mean ± SD. n = 3 mice. Significant differences were determined by Student *t*-test. *p < 0.05.

Our results have revealed that both resistant and susceptible mouse strains are protected against the development of lesions by leishmanization with *L*. *major* plus Pam3CSK4. Our previous work with live parasites and CpG DNA revealed that Th17 responses were required to control vaccinal pathology in C57BL/6 mice [17]. However, the immune response of the BALB/c mice to this vaccine has remained uncharacterized. To investigate whether the CpG DNA-induced Th17 cell expansion is shared with other TLR agonists, we determined the absolute number of cytokine producing CD4 T cells in ears and ear draining lymph nodes of the treated mice at early and late time points. In C57BL/6 mice, both Th17 and Th1 responses at the early time point were enhanced by leishmanization with live parasites and Pam3CSK4 (Fig 5A). This enhanced CD4^+^ T cell response was similar to what was described in our previous work using CpG DNA [17]. In contrast, leishmanization with *L*. *major* plus Pam3CSK4 did not enhance Th17 response in the BALB/c strain, although the number of Th1 IFN-γ expressing cells was higher in the *L*. *major* plus Pam3CSK4 inoculated group (Fig 5D); the immune response in these mice was dominated by Th1 cells, as opposed of what was found in the mice infected with *L*. *major* alone. This trend continued throughout the course of the infection, as demonstrated by the data obtained in the late time point and the Th1/Th2 ratio (Fig 5B, C, E, F). Notably, Th1/Th2 ratio in BALB/c mice indicated that leishmanization with *L*. *major* plus Pam3CSK4 strongly promoted the development of Th1 response. More importantly, both parasite-specific Th1 and Th17 responses were enhanced at sites of infection in C57BL/6 mice infected with *L*. *major* plus Pam3CSK4 at late time point, whereas only Th1 response was enhanced in BALB/c mice (Fig 5G). These data further suggest that the mechanisms underlying protection are different between both mouse strains.

### Pam3CSK4 induces neutrophil influx and IL-17 production at the site of leishmanization

**Fig 6:**
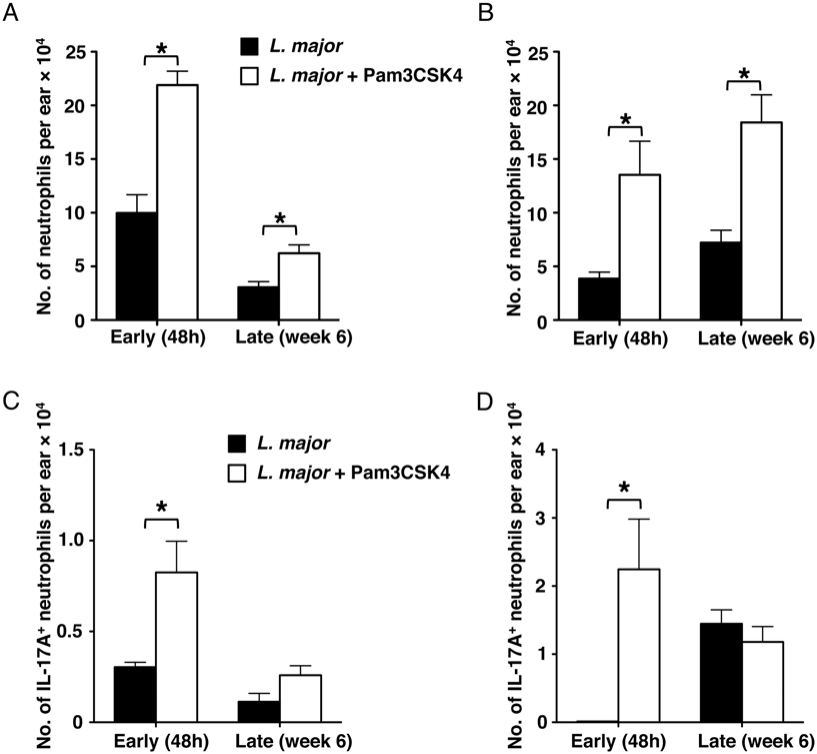
Leishmanization with *L*. *major* + Pam3CSK4 induces neutrophil influx and IL-17 production. Absolute number of Ly-6G^+^ neutrophils shows in (**A**) C57BL/6 and (**B**) BALB/c mice ears. Absolute number of dermal IL-17^+^ Ly-6G^+^ neutrophils determined by flow cytometry in (**C**) C57BL/6 and (**D**) BALB/c mice. Data were collected at day 2, 42 (C57BL/6) and 70 (BALB/c) post injection. Recovered ear cells were retimulated with 25 μg/mL soluble *Leishmania* antigen, 5 ng/mL IL-2 and 10 μg/mL anti-CD28 overnight before performing cytokine staining for IL-17. Each data set was collected from two experiments with similar results. Values represent mean ± SD. n = 3 mice. Significant differences were determined by Student *t*-test. *p < 0.05.

We have reported that, in C57BL/6 mice, vaccination with live parasites and CpG DNA increased the influx of neutrophils to the vaccination site early after vaccination [17]. Similarly, shortly after leishmanization, the number of neutrophils was significantly increased in both C57BL/6 and BALB/c (Fig 6A, B). However, neutrophil numbers were dramatically decreased at the late time point in C57BL/6 mice (Fig 6A). In contrast, despite the lack of pathology, neutrophil numbers remained high in the skin of BALB/c mice received leishmanization with *L*. *major* plus Pam3CSK4. Because other groups have reported increasing amounts of IL-17 production by neutrophils in infected BALB/c mice [29], we assessed the ability of IL-17 production from neutrophils following leishmanization. At the early time point, there was a greater number of IL-17 producing neutrophils infiltrating sites of infection in both mouse strains after leishmanization with *L*. *major* plus Pam3CSK4 (Fig 6C, D).

## Discussion

To date, there is no vaccine against cutaneous leishmaniasis. The failure in translating data from animal models to human disease and a lack of understanding in how protective immune responses and immunological memory are generated and maintained, have been the major impediment in vaccine design [30]. In this paper, we have discovered that leishmanization with live parasites in the presence of the TLR2 agonist Pam3CSK4 prevents the development of lesions in both susceptible and resistant mice, albeit the underlying immunological mechanisms appear to be completely different.

Our work has focused on understanding how live vaccination immunity is modulated by the addition of TLR agonists. In particular, we have extensively characterized immune responses of C57BL/6 mice to vaccination with live parasites plus the TLR9 agonist CpG DNA [14–17,31,32]. We employed this mouse strain because, unlike the susceptible BALB/c mice that succumb to systemic disease by *L*. *major*, infection of C57BL/6 mice replicates all clinical features of human cutaneous leishmaniasis: self-healing lesions [33,34], chronicity [35] and concomitant immunity [36].

The first objective of this study was to validate the immunological mechanism of protection behind live vaccination with CpG DNA, and to investigate whether this mechanism is shared with other TLR agonists. We chose TLR2 because this is the most promiscuous TLR receptor, being able to recognize the most diverse set of pathogen-associated molecular patterns (PAMP). Furthermore, lipophosphoglycan (LPG), a PAMP in *Leishmania*, has been shown to bind to TLR2 and activate NF-κB translocation in a TLR2-dependent manner. This ligation upregulates TLR2 expression and eventually promotes the production of IFN-γ and TNF-α in NK cells [26,27]. Moreover, TLR2 is widely expressed among human leukocytes, which will ensure a very intense response following receptor ligation. More importantly, the higher expression of TLR2 on macrophages is associated with the better disease outcome in cutaneous leishmaniasis patients [37], indicating the clinical relevance of using TLR2 agonists. We have shown that *L*. *major*-infected cells become more sensitive to TLR2 stimulation and increase their proinflammatory response. Our data demonstrate that leishmanization with live parasites plus the TLR2 agonist Pam3CSK4 completely protected mice against the development of lesions, suggesting that TLR2 stimulation also results in enhancing anti-leishmanial immunity. Unexpectedly, we have found that expression of TLR2 in DCs is different between the two strains of mouse. This result is similar to the previous report that showed expression levels of TLR2, TLR4, TLR5 and TLR6 in naïve splenic DCs are higher in BALB/c mice than in C57BL6 mice [38]. The reactivity of DCs in both strains of mouse is also different upon TLR ligand stimulation. Taken together, our data suggest that differences in both expression pattern and reactivity of TLR2 may be associated with susceptibility and resistance to *L*. *major* infection in C57BL/6 and BALB/c mice.

The second objective of our work was to compare the immunological events associated with protection following vaccination of both genetically susceptible and resistant mouse strains, which are characterized by extreme Th2 or Th1 polarization, respectively. The immune responses to cutaneous leishmaniasis in humans lack the strong polarity found in mouse models. Epidemiological data from patients with localized cutaneous leishmaniasis seem to confirm the Th1/Th2 dichotomy shown in mice. Moreover, patients with diffuse cutaneous leishmaniasis display a more predominant Th2 cytokine response. Furthermore, patients with mucosal leishmaniasis show a mixture of Th1 and Th2 cytokines [39]. Thus, the comparative study of the mouse models is important to be able to predict how, and whether, vaccine efficacy studies that employ TLR2 agonists would translate to human vaccines.

Our data showing that leishmanization with parasites plus Pam3CSK4 protects both C57BL/6 and BALB/c mice against lesions are very promising, and point towards the feasibility of the use of TLR2 agonists as immune adjuvants against leishmaniasis. However, our studies have also revealed that the mechanism underlying protection is very different between the two mouse strains. Firstly, in contrast to C57BL/6 mice, parasite burden was decreased in BALB/c mice that received leishmanization with parasites plus Pam3CSK4 at the early time point, indicating parasite killing in BALB/c mice was enhanced at the early time point. This is highly unlikely to be caused by the cytotoxicity of Pam3CSK4 on parasites before inoculation, as we did not observed significant difference of the viability of *L*. *major* in all our *in vitro* experiments. In addition, unchanged parasite burden in C57BL/6 mice at the same time point further rules out the possibility of cytotoxicity of Pam3CSK4 on parasites. We speculate that the early parasite killing enhanced by the addition of Pam3CSK4 in BALB/c mice relies on high expression of TLR2 in immune cells. Indeed, compared to C57BL/6 mice, macrophages in BALB/c mice produce more IL-12 in responding to Lm+Pam3CSK4 (See Fig 3B and D). This indicates the enhanced activation of macrophages, which may lead to increase the production of nitric oxide, a toxic to *L*. *major*. Nevertheless, other effects on tissue that may be caused by inoculation of Pam3CSK4 need to be further investigated. Secondly, C57BL/6 mice, as we demonstrated before, develop a strong Th17 response following vaccination with TLR agonists. However, this effector population did not expand in treated BALB/c mice; in these animals, protection appears to be mediated by the enhanced Th1 response. The enhanced Th1 response in both strains will lead to the production of nitric oxide by activated macrophages at sites of infection, which mediates killing of parasites. A recent study by Pandey et al. showed that treating infected mice with pegylated bisacycloxypropylcysteine (BPPcysMPEG), a TLR2-TLR6 ligand, is capable of conferring protection against *L*. *major* infection in BALB/c mice [40]. Importantly, administration of BPPcysMPEG after immunization with fixed *L*. *major* induced protection against challenge infection. Interestingly, this study showed that treatment of Pam3CSK4 failed to reduce parasite burden. The different outcomes compared to our results could be because of the timing of TLR2 agonist administration. After three days of infection, parasites have established the infection, which strongly suppresses the activation of TLR1-TLR2 signaling in macrophages. This is consistent with our *in vitro* data indicating that *L*. *major* infection in BALB/c macrophages inhibits production of IL-12 induced by Pam3CSK4. Moreover, the different infection route (subcutaneous vs intradermal) and infection dose may also contribute to the outcomes of infection [34,41].

Another remarkable difference between the two strains was the sustained neutrophil influx in BALB/c, but not in C57BL/6 mice. The role of the neutrophil in leishmaniasis is not well understood because it varies depending on the species of *Leishmania* and the animal models employed. Studies in the C57BL/6 mice have shown that neutrophils may promote infection by harboring parasites [42]. Conversely, others have revealed that neutrophils contribute to parasite killing [43]. Consistent with our results, neutrophil influx has been associated with resistance in *L*. *amazonensis* murine models [44,45]. Finally, neutrophils appear to be required for protective responses in *L*. *braziliensis* [46]. Our data also uncovered an interesting outcome of leishmanization which is at early time point, large numbers of neutrophils infiltrate to sites of infection in both C57BL/6 and BALB/c mice that were inoculated with live parasites plus Pam3CSK4. This may be associated with the reduced parasite burden and pathology. Moreover, neutrophil infiltration induced by the additional Pam3CSK4 is sustained in BALB/c mice, which may be due to the specific effect of Pam3CSK4 in this cell type only in the susceptible strain. Some studies have already shown the distinct phenotypes of neutrophils expressing different TLRs in both resistant and susceptible mice during *L*. *major* infection [47]. Differential expression of TLRs by neutrophils may cause the diverse responses to TLRs agonist and thus influence the development of *L*. *major* specific immune response in our leishmanization approach.

Notably, leishmanization with *L*. *major* and Pam3CSK4 induces the production of IL-17 from neutrophils in both strains of mouse. The role of IL-17 in leishmaniasis is controversial. In BALB/c mice, IL-17 promotes progression of disease [29]. However, it has been associated with protection against the infection of *Leishmania donovani* and in our previous vaccine model [17,48]. In our current model, *L*. *major* plus Pam3CSK4 enhance Th1 and Th17 responses, which are associated with protection in C57BL/6 mice. In contrast, only Th1 but not Th17 response seems to be required for protection in BALB/c mice. Therefore, we speculate that the outcome of production of IL-17 depends on background of the host. Moreover, IL-17 confers protection by actively recruiting neutrophils. A recent study has revealed autocrine IL-17 activity in mouse neutrophils [49], indicating that neutrophil-derived IL-17 may contribute to the sustained influx of neutrophils in BALB/c mice. The function of IL-17 driven from neutrophils in *L*. *major* infection requires further investigation.

Several studies have shown that administration of TLR2 agonists confers protective immunity against *Leishmania* [40,50]. IL-12 has been shown to be essential to sustain the generation of memory T cells, which provides long-term protective immunity against *L*. *major* [51,52]. As a potent IL-12 inducer, inoculation of Pam3CSK4 stimulates large amount of IL-12 from dermal DCs and macrophages at sites of infection. Therefore, we speculate that leishmanization with *L*. *major* and Pam3CSK4 is highly likely to be able to induce protective immunity.

Our findings are relevant because they reveal the complexity and the difficulty to achieve vaccine protection: by exclusively taking into account the C57BL/6 data, we would have concluded that enhancing Th17 response is necessary to protect against leishmanial challenge. However, the effectiveness of Th17 response depends on the individual. Understanding the factors that regulate parasite persistence and its role in maintenance of immunologic memory in cutaneous leishmaniasis is critical for development of effective vaccines and vaccination strategies against the disease, and may explain why vaccination strategies have not translated very well from mouse to human.
